# Supplementary material for: SGK regulates pH increase and cyclin B–Cdk1 activation to resume meiosis in starfish ovarian oocytes
Source: J Cell Biol. 2019 Sep 19;218(11):3612–29. doi: 10.1083/jcb.201812133 (PMC6829648; doi:10.1083/jcb.201812133)
Supplement: Supplemental Materials (PDF) [file JCB_201812133_sm.pdf]

Supplemental material

Hosoda et al., https://doi.org/10.1083/jcb.201812133

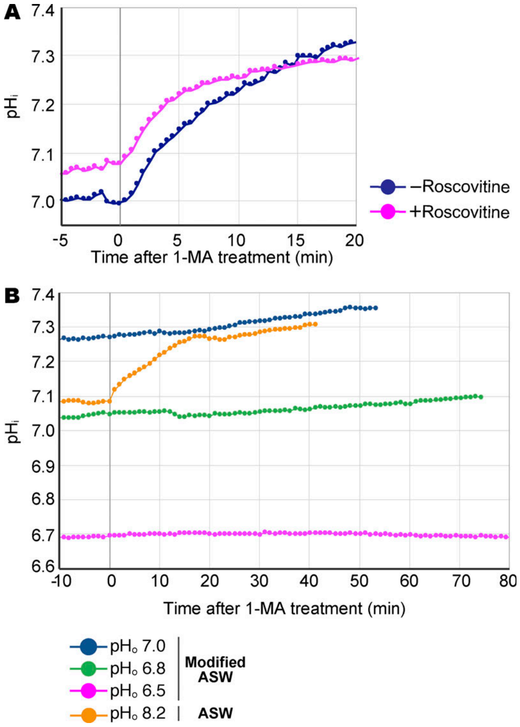

Figure S1. **pH$_i$ values in 1-MA–treated oocytes were measured in ASW with roscovitine or in modified ASW for pH$_i$ clamping. (A)** pH$_i$ increase soon after 1-MA stimulus is independent of cyclin B–Cdk1 activation. Unstimulated oocytes were injected with BCECF-dextran, incubated in the presence or absence of the selective Cdk inhibitor roscovitine for 1 h, and then stimulated with 1-MA. The fluorescence intensity ratio was measured every 30 s before and after 1-MA addition. Then, pH$_i$ was calculated based on the averaged fluorescence intensity ratio of 11 and 8 oocytes in the presence and absence of roscovitine, respectively, and plotted on the indicated graph. This result is a representative of two independent experiments. pH$_i$ was elevated after 1-MA stimulus even in the presence of roscovitine, as in control oocytes. **(B)** pH$_i$ values were clamped in modified ASW containing CH_3COONH_4. Unstimulated oocytes were injected with BCECF-dextran and incubated for 20 min in ASW or modified ASW with pH$_o$ values of 6.5, 6.8, and 7.0 to clamp the pH$_i$ at ~6.7, 7.0, or 7.2, respectively. The fluorescence intensity ratio was measured each minute before and after 1-MA addition. The pH$_i$ values were calculated from the averaged fluorescence intensity ratios of 8–10 oocytes and plotted on the indicated graph. This result is representative of two independent experiments. The pH$_i$ increased rapidly after 1-MA stimulation in ASW but not in modified ASW.

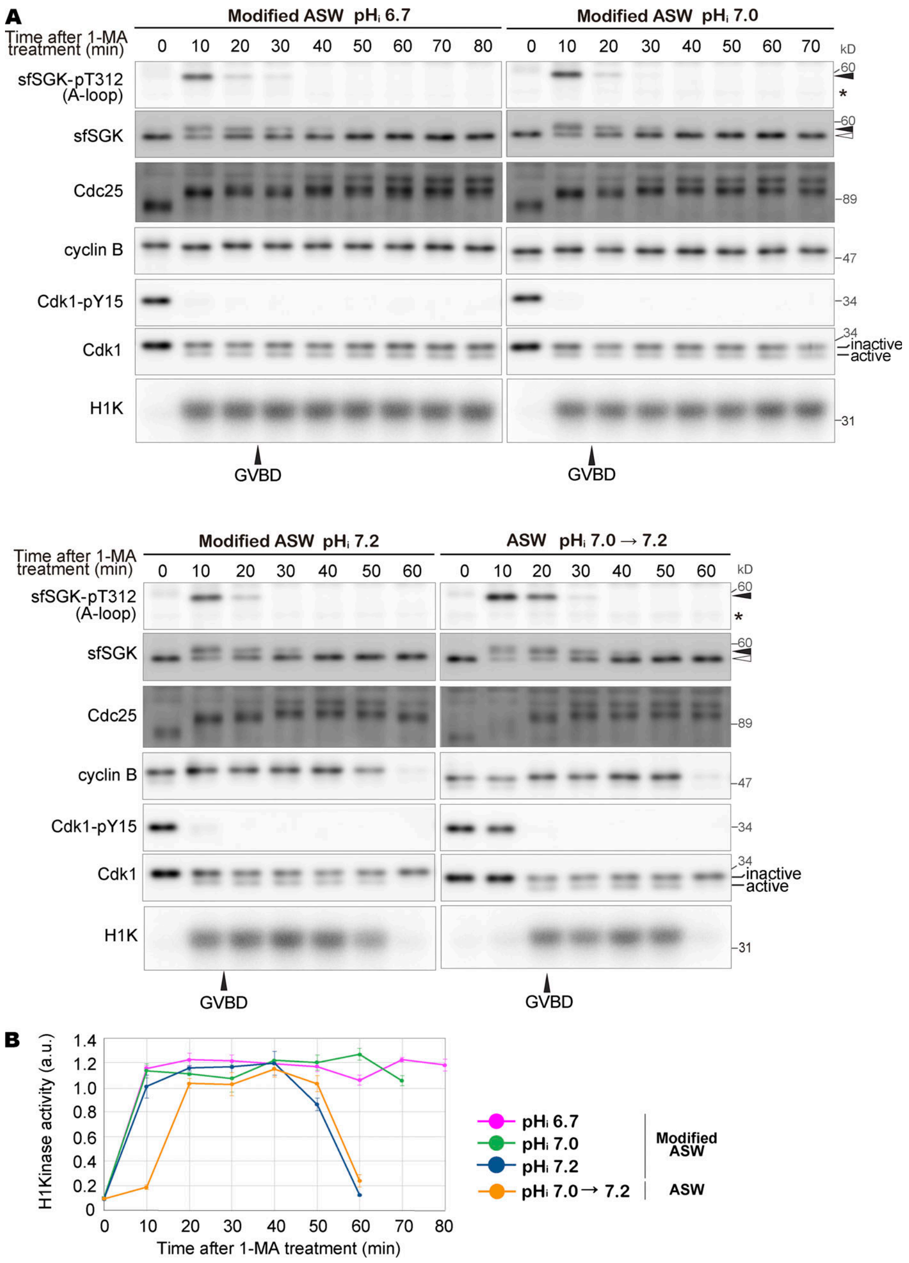

Figure S2. **H1 kinase activity was measured in oocytes after 1-MA stimulation at various clamped pH_i values. (A and B)** Unstimulated oocytes were incubated in modified ASW to clamp the pH_i at ~6.7, 7.0, or 7.2 or in ASW as a control. They were then stimulated with 1-MA. Oocytes were collected at the indicated time points and subjected to immunoblotting (A) or used for H1 kinase assays to measure the cyclin B–Cdk1 activity. In the H1 kinase assay, [γ-^{32}P] ATP incorporation into histone H1 was detected via autoradiography (A) and quantified via liquid scintillation counting (B). The times to GVBD after 1-MA stimulation were ~20 min in ASW and ~15, ~14, and ~21 min at clamped pH_i values of 7.2, 7.0, and 6.7, respectively. Closed and open arrowheads in A indicate the positions of the upper and lower sfSGK bands, respectively. Asterisks in A indicate nonspecific bands. The graph in B indicates levels of H1 kinase activity relative to that in oocytes treated with 1-MA for 30 min in ASW (means ± SE of three independent experiments). The levels of cyclin B–Cdk1 activity were comparable among these conditions. In ASW or at a clamped pH_i of 7.2, cyclin B–Cdk1 was inactivated via cyclin B degradation 60 min after 1-MA stimulation. By contrast, when the pH_i was clamped at 7.0 or 6.7, the cyclin B protein level and cyclin B–Cdk1 activity were maintained for a longer time.

https://doi.org/10.1083/jcb.201812133

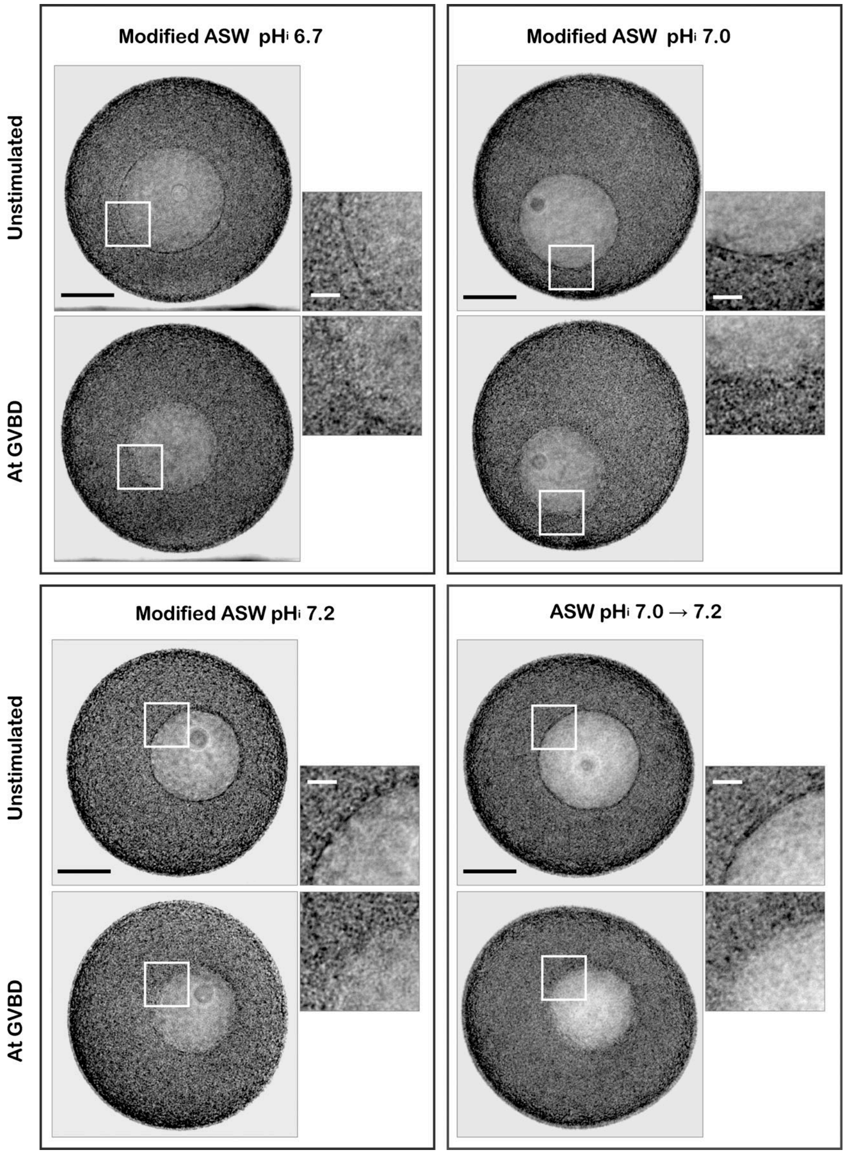

Figure S3. **Morphology of GVBD is normal at various clamped pH_i values.** To clamp the pH_i at ∼6.7, 7.0, and 7.2, unstimulated oocytes were incubated with modified ASW for 20 min. As a control, oocytes were incubated in ASW for 20 min, in which pH_i is ∼7.0 and increases to ∼7.2 after 1-MA treatment. Nomarski DIC images of unstimulated oocytes and 1-MA–stimulated oocytes at GVBD were captured. Left column: Images of whole oocytes. Right column: Enlarged view of the rim of GV corresponding to the white rectangles. The black scale bar in the left column represents 50 µm, and the white scale bar in the right column represents 10 µm. Results in all panels are representative of two independent experiments. In ASW, the rim of GV in unstimulated oocytes looked like a clear line. Cytoplasmic granules started to invade into the inner GV area at GVBD. The rims of GV in unstimulated oocytes and at GVBD at all clamped pH_i values were morphologically indistinguishable from those in ASW.

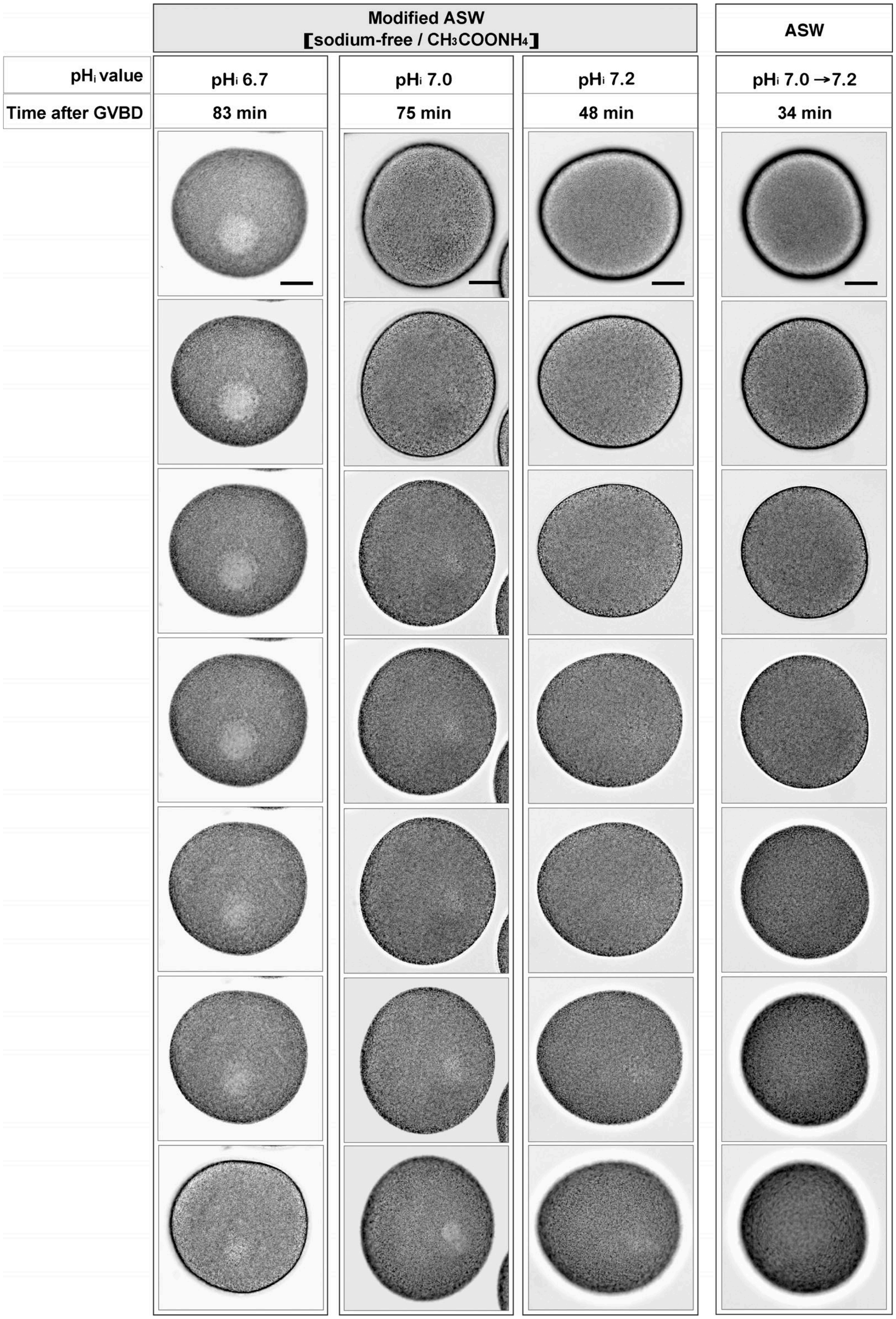

Figure S4. **Cytoplasmic granule invasion into the GV region was drastically impaired at a clamped pH_i of 6.7.** Unstimulated oocytes were incubated in modified ASW to clamp pH_i at ~6.7, 7.0, and 7.2 or in ASW as a control, and then treated with 1-MA. Time-lapse DIC imaging was performed (see Videos 1, 2, 3, and 4). Then, to ascertain whether cytoplasmic granule invasion was completed, Z-stacks of these oocytes were taken by DIC microscopy at the indicated times after GVBD. Images from each Z-stack are shown. The scale bars represent 50 µm. Cytoplasmic granules were homogenously distributed throughout oocytes in ASW and oocytes at a clamped pH_i of 7.2. At a clamped pH_i of 7.0, a trace of the inner nuclear region was observed. At a pH_i of 6.7, a large part of the inner nuclear region had not been invaded by the granules.

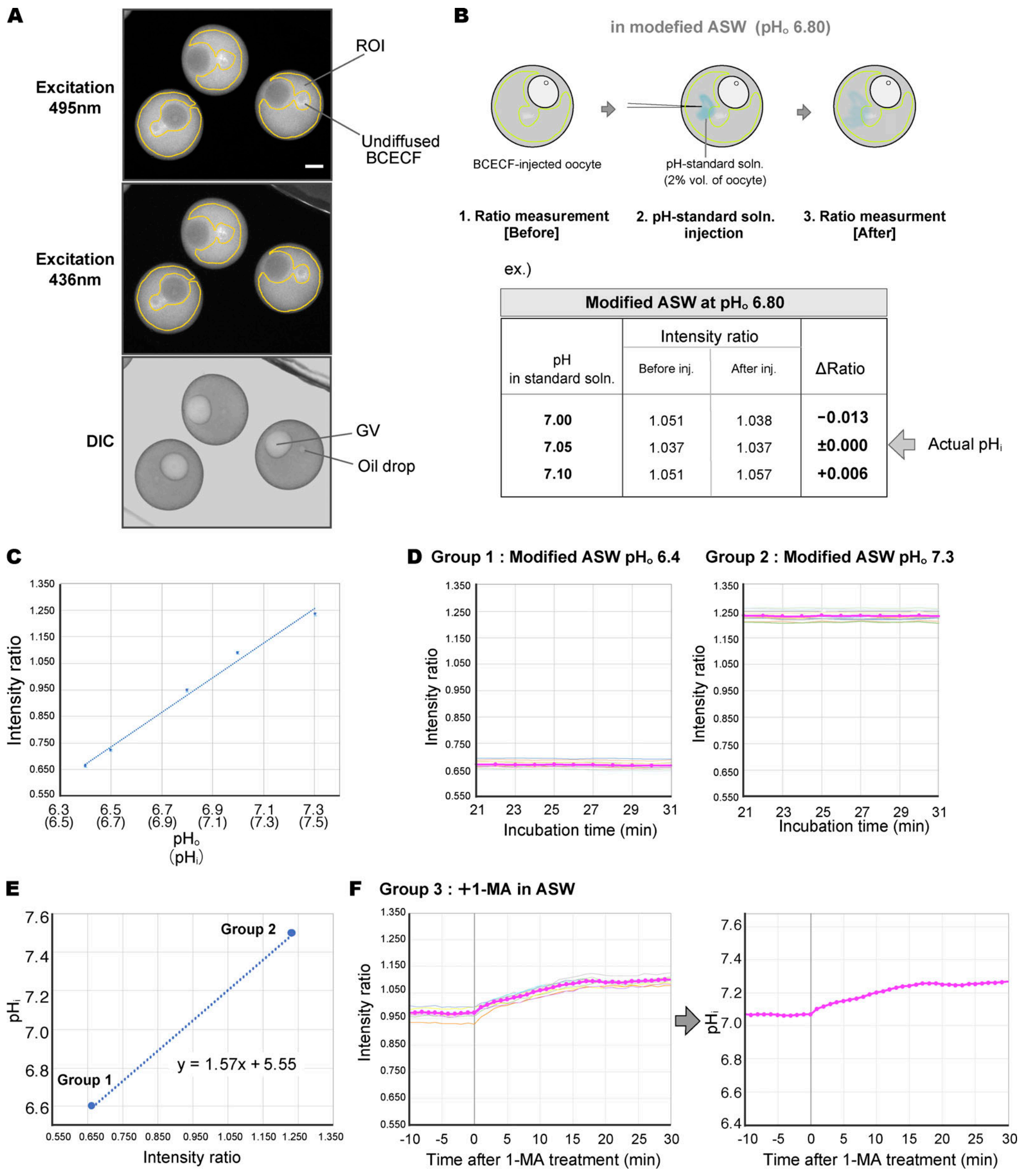

Figure S5. **pH_i measurement using BCECF-dextran. (A)** An example ROI for fluorescence ratio measurement is shown. The upper and middle panels show typical fluorescence images (535 nm emission) in BCECF-injected unstimulated oocytes following excitation at 495 nm and at 436 nm, respectively. ROIs (yellow lines) can be freely drawn on the images using HCImage software, and they were defined to surround a cytoplasmic region excluding the GV region. An oil drop introduced along with the BCECF as a mark of the microinjection can be observed in each injected oocyte. Although most of the injected BCECF was diffusely distributed throughout the cytoplasm after injection, a small fraction usually stayed at the injection site, visible in the fluorescent images as a small brighter region near the oil drop. The bright regions were excluded from the ROIs. Bar, 50 µm **(B)** An example of pH_i determination under steady-state conditions. In this example, unstimulated oocytes were injected with BCECF-dextran and incubated in modified ASW with a pH of 6.8. Thereafter, pH-standard solutions with the indicated pH values were injected, and the fluorescence ratios were measured. In this case, the ratio decreased immediately after injection of the pH 7.00-standard solution (soln.), indicating that the pH_i before injection (inj.) of the standard solution was higher than pH 7.00. The ratio did not change after injection of the pH 7.05-standard solution, indicating that the pH_i before injection of the standard solution was equal to ~7.05. Consistently, the ratio increased immediately after injection of the pH 7.10-standard solution. Together, these results indicate that the pH_i was ~7.05 in this case. **(C)** The fluorescence ratio and pH_i values show a linear correlation at least within a range between pH_i 6.6 and pH_i 7.5. Unstimulated oocytes were injected with BCECF-dextran and incubated in modified ASW with pH_o values of 6.4, 6.5, 6.8, 7.0, and 7.3 to clamp the pH_i. The fluorescence ratios (mean ± SE of 8–11 oocytes) were measured and plotted against the pH_o values. The pH_i values, which were ~0.2 higher on average compared with pH_o values of the modified ASW (Moriwaki et al., 2013), are indicated in parentheses. **(D–F)** An example of a time course pH_i measurement is shown. To determine the pH_i values in a time course experiment, oocytes were injected with BCECF-dextran and separated into three groups. **(D)** The two groups were incubated in modified ASW at pH 6.4 (group 1) and pH 7.3 (group 2) to clamp the pH_i at ~6.6 and 7.5, respectively. The fluorescence ratios were measured in 10 or 11 oocytes (thin lines), providing average ratios corresponding to pH_i values of 6.6 and 7.5 (thick magenta lines). **(E)** The average ratios were plotted against the pH_i. A linear function was calculated in E and then used as a standard function to convert the measured fluorescent ratios to the pH_i values shown in F (see below). The third group was subjected to an experimental condition. In this example, oocytes were treated with 1-MA in ASW. **(F)** Time-lapse recordings of the fluorescence ratios were obtained from nine oocytes (left graph, thin lines). Averaged ratios (left graph, thick line) were converted to pH_i values (right graph) using the standard function obtained in E.

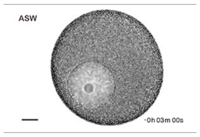

Video 1. **GVBD of oocytes in ASW.** Unstimulated oocytes were incubated in ASW as a control. Time-lapse DIC imaging was performed every 10 s before and after 1-MA treatment. The video starts at 3 min before 1-MA treatment. “h: m: s” in frames represents hour, minutes, and seconds after 1-MA treatment. All images were acquired by focusing the microscope on the equatorial plane of GV region. Focus was not changed during time-lapse imaging. This video runs for 45 min. Selected images from the image sequence are shown in Fig. 5 C. Bar, 25 µm.

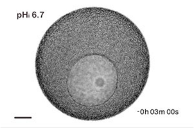

Video 2. **GVBD of oocytes in modified ASW for clamping pHi at 6.7.** Unstimulated oocytes were incubated in modified ASW to clamp pH_i at 6.7. Time-lapse DIC imaging was performed every 10 s before and after 1-MA treatment. The video starts at 3 min before 1-MA treatment. “h: m: s” in frames represents hour, minutes, and seconds after 1-MA treatment. All images were acquired by focusing the microscope on the equatorial plane of GV region. When the equatorial plane was moved out of focus during the time-lapse imaging, the focus was returned on the equatorial plane (0:04:30, 0:20:40, 0:24:00, 0:27:40, 0:33:20, 0:42:00, 0:46:10, 0:57:50, 01:02:50, and 01:07:30). This video runs for 1 h 20 min. Selected images from the image sequence are shown in Fig. 5 C. Bar, 25 µm.

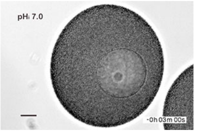

Video 3. **GVBD of oocytes in modified ASW for clamping pHi at 7.0.** Unstimulated oocytes were incubated in modified ASW to clamp pH_i at 7.0. Time-lapse DIC imaging was performed every 10 s before and after 1-MA treatment. The video starts at 3 min before 1-MA treatment. “h: m: s” in frames represents hour, minutes, and seconds after 1-MA treatment. All images were acquired by focusing the microscope on the equatorial plane of GV region. Focus was not changed during time-lapse imaging. This video runs for 1 h 20 min. Selected images from the image sequence are shown in Fig. 5 C. Bar, 25 µm.

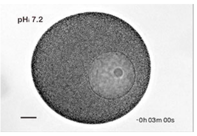

Video 4. **GVBD of oocytes in modified ASW for clamping pHi at 7.2.** Unstimulated oocytes were incubated in modified ASW to clamp pH_i at 7.2. Time-lapse DIC imaging was performed every 10 s before and after 1-MA treatment. The video starts at 3 min before 1-MA treatment. “h: m: s” in frames represents hour, minutes, and seconds after 1-MA treatment. All images were acquired by focusing the microscope on the equatorial plane of GV region. Focus was not changed during time-lapse imaging. This video runs for 55 min. Selected images from the image sequence are shown in Fig. 5 C. Bar, 25 µm.

Reference

Moriwaki, K., T. Nakagawa, F. Nakaya, N. Hirohashi, and K. Chiba. 2013. Arrest at metaphase of meiosis I in starfish oocytes in the ovary is maintained by high CO_2 and low O_2 concentrations in extracellular fluid. *Zool. Sci.* 30:975–984. https://doi.org/10.2108/zsj.30.975
